# E-Research Institutional Cloud Architecture (ERICA): An Orchestration Meta-Framework for Establishing Trusted Research Environments Using Public Cloud Computing

**DOI:** 10.23889/ijpds.v6i1.3373

**Published:** 2026-05-20

**Authors:** Timothy R Churches, Richard Green, Phi Bang Nguyen, Md Shajedur Rahman Shawon, Louisa R Jorm

**Affiliations:** 1 Ingham Institute for Applied Medical Research, Syndey, Australia; 2 University of New South Wales, Sydney, Australia

**Keywords:** Trusted Research Environment, cloud computing, sensitive data, five safes

## Abstract

**Introduction:**

The E-Research Institutional Cloud Architecture (ERICA) is a code-driven orchestration framework that automates the configuration and management of Amazon Web Services (AWS) resources to provide trusted research environments (TREs) for sensitive data. Independent ERICA TREs are now operational in universities and government agencies. The framework was developed by the University of New South Wales, Australia, with support from the Australian Research Data Commons.

**Objectives:**

ERICA was designed to overcome the limitations of traditional on-premise TREs by providing secure, scalable, and flexible cloud environments that protect privacy while enabling advanced, data-intensive research.

**Approach:**

Using an infrastructure-as-code model, ERICA delivers consistent, reliable, and error-free setup of Project Spaces. It integrates robust security features, including encryption-at-rest and in transit, and multi-factor authentication. Hosted in AWS onshore data centres, ERICA ensures data sovereignty while supporting diverse operating systems and high-performance computing configurations. The architecture also allows rapid deployment of new AWS services, including generative AI tools, within research workspaces.

**Discussion:**

ERICA implements the Five Safes framework—covering safe projects, people, data, settings, and outputs—to ensure compliance and secure research. Its modular architecture enables multiple independent TREs, each governed by host-institution policies and capable of supporting hundreds of Project Spaces. This flexibility allows replication across any jurisdiction with AWS public cloud infrastructure. However, reliance on AWS introduces challenges, including charges in US dollars and delayed rollout of new services in smaller regions.

**Conclusions:**

ERICA represents a step change in providing privacy-by-design cloud infrastructure for sensitive, data-intensive research. By combining strong governance with the scalability of AWS, it enables researchers to work securely with large, complex datasets while rapidly adopting cutting-edge analytical tools. ERICA TREs offer a replicable, future-proof model for supporting secure research at scale.

## Introduction

Contemporary research across domains such as health, socialscience and genomics increasingly relies on the analysis of large-scale, sensitive personal data, including linked adminis-trative records, clinical data, and -omics datasets. Analyses often involve advanced methods such as machine learning, natural language processing, and large-scale simulation modelling, which require significant computational power. This brings a fundamental challenge: how to enable meaningful, data-intensive research while upholding the highest standards of privacy, security and ethical data governance [1].

Trusted Research Environments (TREs) have emerged as a key solution—secure, purpose-built platforms that embed ‘privacy-by-design’ principles, enabling researchers to work safely with sensitive datasets under tightly controlled conditions [2]. TREs are now widely recognised as essential infrastructure for delivering high-impact research without compromising data integrity or public trust [3]. In Australia, the development and adoption of TREs has been identified as a national priority, particularly to support secure access and linkage of health and population data at scale [4].

The E-Research Institutional Cloud Architecture (ERICA) platform delivers a secure, flexible, and scalable cloud environment purpose-built for researchers working with sensitive data. Initially developed and funded by the University of New South Wales (UNSW), ERICA became operational in 2018 and has since received additional support from the Australian Research Data Commons (ARDC) to expand its capabilities and adoption.

ERICA is a cloud orchestration meta-framework—built as infrastructure-as-code—that offers substantial advantages over traditional fixed-infrastructure data enclaves. By harnessing the scalability and security of Amazon Web Services (AWS), ERICA supports a broad spectrum of computational needs, from standard virtual desktops to high-performance compute, all within tightly isolated, policy-driven Project Spaces hosted inside the ERICA instance. Its code-driven architecture enables the efficient creation of fully independent “cloned” instances, allowing individual organisations to deploy their own ERICA TREs while maintaining full governance control. Today, the ERICA underpins a growing ecosystem of TREs across universities and government agencies in Australia, uniting them through a common technical foundation while enabling fully independent governance and operational control tailored to each host institution.

The aim of this paper is to describe how ERICA’s code-driven, privacy-by-design framework implements the Five Safes principles (safe projects, people, data, settings, and outputs) [5] to establish trusted research environments using public cloud infrastructure along with its operating model, technical architecture, data governance approach, and some notable research use-cases.

## Approaches

### Secure AWS Cloud Infrastructure and Regulatory Compliance

ERICA consists of a set of custom web applications and backend services that orchestrate resources in the AWS public cloud. All data and computing for the UNSW ERICA instance are contained within AWS onshore data centres in Sydney, ensuring data sovereignty (no sensitive data ever leaves the country). AWS’s infrastructure meets stringent security requirements and is accredited under global standards including ISO 27001 (information security) and related cloud security standards. Most AWS services used by ERICA are also certified under the Australian Information Security Registered Assessors Program (IRAP) [6], meaning they are approved for use with highly sensitive data. This compliance foundation allows ERICA to inherit robust physical and cloud security controls from AWS.

By building on AWS, ERICA benefits from a highly reliable and scalable infrastructure. Data within an ERICA instance are replicated across multiple AWS availability zones (geographically separate data centres within the same region) for resilience. AWS provides security features including encryption-at-rest, comprehensive sub-network isolation, and immutable monitoring logs for every aspect of an ERICA instance. ERICA leverages these and other features to enforce strong cybersecurity (detailed later under the Five Safes implementation). Additionally, because ERICA uses standard AWS services, new cloud computing capabilities – for example, advanced machine learning or generative AI services – can be integrated into the environment relatively quickly once they become available and approved for use. This ensures that ERICA-based TREs can evolve to utilise cutting-edge tools while continuing to implement a secure framework.

ERICA instances operate within a complex national regulatory environment shaped by both federal and state legislation. At the federal level, the Privacy Act 1988, overseen by the Office of the Australian Information Commissioner (OAIC), governs the handling of personal information [7]. In New South Wales (NSW), ERICA is accredited under eHealth NSW’s Privacy and Security Assessment Framework (PSAF) [8] to hold NSW Health data, demonstrating compliance with state-specific governance protocols. However, hosting Commonwealth datasets such as the Medicare Benefits Schedule (MBS) and Pharmaceutical Benefits Scheme (PBS) requires additional accreditation under the under the National DATA Scheme administered by the Office of the National Data Commissioner [9]. ERICA is actively working towards achieving this accreditation to enable secure access to these high-value national datasets within its trusted research environments.

### Code-Driven Orchestration Framework

A core innovation of ERICA is its infrastructure-as-code orchestration (IaC) [10], which automates the setup and management of the TRE. Instead of manually configuring cloud resources, system administrators deploy ERICA using code scripts (AWS CloudFormation and other IaC tools) that consistently instantiate the required AWS components and security controls – both the infrastructure components for the overall ERICA TRE instance, as well as individual, isolated and self-contained Project Spaces withing that ERICA instance.This code-driven approach ensures each Project Space and service is configured in a precise, repeatable manner, minimising the risk of human error and misconfiguration. Every aspect of the environment – virtual network settings, storage encryption, user workspaces, audit logging, etc. – is defined in code and can be tested and version-controlled. As a result, new ERICA instances or updates can be rolled out reliably using standard DevOps (Development and Operations) IT workflows, and best practices are enforced uniformly across all instances.

To complement the backend automation, ERICA provides an intuitive web-based interface (the ERICA Hub) for configuring projects and managing data ingress/egress. Researchers and data custodians interact with ERICA through this interface to request project setups, upload or download approved files, and access virtual analysis workstations. This point-and-click front end makes it feasible for users with limited IT expertise to utilise the platform, while the underlying orchestration translates their requests into secure AWS resource deployments behind the scenes using automation which mitigates against human configuration errors compromising security.

As mentioned, ERICA’s architecture allows multiple independent instances of the platform to be deployed for different organisations while maintaining strict isolation between them. Each instance (or “clone”) operates under its host institution’s AWS account and governance – for example, UNSW operates one instance and the NSW Government’s Data Analytics Centre operates another, with no shared administrative personnel – only the orchestration code used to establish each instance is shared. Each ERICA instance can support up to 255 Project Spaces – essentially isolated virtual research environments – for different projects or teams. Project Spaces within an instance are completely segregated from one another (network isolation and access controls), and likewise there is no connectivity or data sharing across different ERICA instances. This multi-instance model, enabled by the common code base, lets each organisation run its own TRE on AWS infrastructure under its control, yet benefit from the shared development, enhancements, and community of practice around ERICA. It embodies a “do once, use many times” philosophy: improvements to the core ERICA code (e.g. new features or security patches) can be propagated to all instances, avoiding duplicate development effort and promoting standardisation.

### Governance Framework

**Figure 1:**
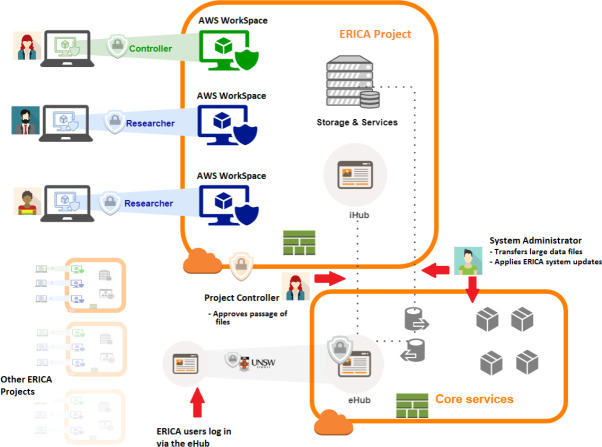
Structure and Management of ERICA Project Spaces

ERICA operates under a clearly defined governance structure that ensures role-based accountability, data security, and institutional compliance (Figure 1). Each instance is led by an ERICA Director, responsible for strategic oversight and policy alignment, and supported by a System Administrator who manages technical operations. An ERICA Manager may also be appointed to coordinate user support, training, and administrative workflows.

Within each Project Space, governance roles are explicitly assigned. The Chief Investigator holds ultimate responsibility for project conduct and may serve as the Project Controller, approving all data and file ingress and egress. A Project Manager, where designated, oversees research team compliance and documentation, while Project Researchers conduct analysis within access constraints defined by project approvals. Data Custodians—often external to the research team—may provide data and act as advisers or Project Controllers to ensure appropriate use. This structured role hierarchy aligns with institutional data stewardship models, ensuring responsibilities for legal compliance, data quality, and breach notification are clearly delineated. Comprehensive logging of user actions, administrative tasks, and all data and file movements in and out of each Project Space enables traceability and accountability.

Governance is further strengthened through regular inter-institutional forums, led by UNSW, where policies are reviewed, platform updates are coordinated, and shared standards are refined. This collaborative model ensures ERICA’s governance remains responsive to emerging needs while upholding the confidence of data custodians, ethics committees, and research teams.

### Operational Model and Sustainability

ERICA adopts a federated operational model, enabling each institution to independently deploy and manage its own instance within a shared technical and governance framework. Originally developed and maintained by UNSW, the platform is made available through licensing or collaboration agreements that support ongoing development and implementation.

Across its operational lifetime, ERICA has been adopted by four Australian institutions (one university and three government agencies). The UNSW ERICA instance currently supports more than 45 Project Spaces and approximately 140 registered users.

Each institution hosts ERICA within its own AWS tenancy and assumes responsibility for local operations, including user management and policy enforcement. UNSW provides second-line technical support and coordinates platform-wide enhancements. This distributed model safeguards data sovereignty while facilitating shared innovation and cost efficiency.

ERICA is not currently open source, and intellectual property is retained by UNSW. However, licensing is available to both Australian and international organisations under bespoke agreements that include access to code, implementation support, and ongoing updates.

Sustainability is underpinned by pooled licensing fees, in-kind contributions, and a collaborative ecosystem that shares training materials, standard procedures, and feature updates. Cost control mechanisms—such as automated resource scaling and user-configurable compute options—support financial viability on commercial cloud infrastructure.

In response to user feedback, the model continues to evolve, exploring microservice architecture, national research infrastructure integration, and potential centralised service delivery.

### Implementation of the Five Safes Framework

**Figure 2:**
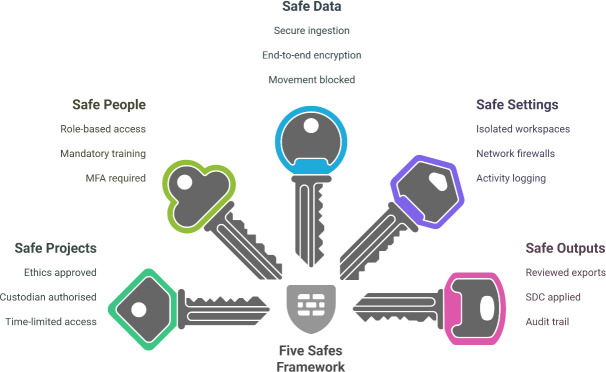
Overview of ERICA’s Implementation of the Five Safes Framework

To ensure a comprehensive privacy-by-design approach, ERICA was built with the Five Safes framework in mind [5] (Figure 2). This framework covers safe projects, safe people, safe data, safe settings, and safe outputs – together addressing the key dimensions needed for a TRE. ERICA is primarily a configurable platform that provides technical enforcement of key controls—such as isolation, access restriction, and audit logging—roles, responsibilities, and institutional governance arrangements (e.g. who performs disclosure control) are defined outside the ERICA codebase. Below, we detail how ERICA’s design and workflows supports implementation of the Five Safes principles.

### Safe Projects

Safe projects are ensured by requiring that all research projects using ERICA adhere to stringent ethical and governance requirements set by the host institution and data providers. Before a Project Space is provisioned in ERICA, the research project must have all necessary ethics committee approvals and data custodian agreements in place. For example, in the UNSW ERICA instance, all projects must provide evidence of Human Research Ethics Committee (HREC) approval [11] that specifically names ERICA as the secure data storage and analysis facility and specifically lists all datasets that will be used. Each Project Space is created for a defined project and has an expiration or review date aligned with the project’s ethics approval—when a project concludes, its Project Space is archived or terminated following a controlled process.

### Safe People

Safe people refers to ensuring that only appropriately authorised and trained individuals can access the relevant Project Spaces and the data they contain in an ERICA instance. ERICA enforces this through clearly defined user roles and stringent authentication. Each ERICA instance has its own Microsoft Active Directory to manage user identities, accounts, and groups. Users are assigned role-based accounts such as System Administrator, Data Custodian, Project Chief Investigator, Project Controller, Project Manager, or Project Researcher. Each role carries specific permissions within the platform. For example, a Project Researcher can log into a secure workspace and perform analyses, but cannot directly import or export data without approval; a Project Controller (often a senior researcher or data custodian delegate) has authority to approve or deny data ingress/egress for their project; and a System Administrator manages the technical environment but cannot see the content of the research data. All users must complete comprehensive online training (including modules on privacy, ethics, information security, and Statistical Disclosure Control) and pass an online exam before they are granted access. This training requirement ensures that researchers understand their responsibilities in handling sensitive data. User accounts are time-limited (e.g. 12-month validity) and must be renewed with approval, preventing lingering access. Multi-factor authentication (MFA) is mandatory for all user logins, adding an extra layer of identity verification. Collectively, these measures ensure that only authorised and appropriately trained personnel (“safe people”) can access an ERICA Project Space (and the ERICA eHub, described below), and that they operate under role-based restrictions aligned with the principle of least privilege.

### Safe Data

**Figure 3:**
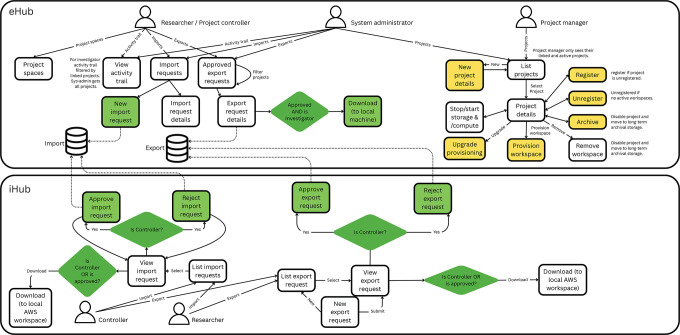
Architecture and Information Technology of ERICA Platform Showing Secure Movement of Data

Safe data means the data used is appropriate and protected throughout its lifecycle in the platform. All data that enters an ERICA Project Space must be approved by the data’s custodian and relevant Project Controller (Figure 3).

Data custodians (the organisations responsible for the source data) typically perform an initial review and then upload the datasets. This is done through an ingress system called the ERICA Hub (or eHub), which provides an upload interface or via a secure transfer to an encrypted AWS S3 bucket designated for that project. Once inside ERICA, all research data is encrypted at rest and in transit. AWS Key Management Service handles encryption keys, and all storage volumes (EBS disks attached to virtual workspaces, S3 buckets, databases, etc.) are encrypted with strong algorithms. Data in transit within the environment uses secure protocols (e.g. HTTPS/TLS1.3 for web access, LDAPS for directory services, secure copy for file transfer, and encrypted SMB/CIFS for any network file shares). The architecture is engineered such that researchers cannot bypass controlled channels: the only way to bring data in or out is through the approved Hub mechanism. All other potential pathways (USB, email, internet uploads and downloads, clipboard transfer, even extrenal DNS queries) are blocked by multiple redundant layers of network rules and system policies. This ensures that unapproved data movements are impossible, greatly reducing the risk of data leakage. By default, all data in ERICA is treated as highly sensitive and thus subject to these architectural constraints, thus minimising risks from inadvertent misclassification of the sensitivity of individual files or other data stores.

### Safe Settings

Safe settings are achieved in ERICA by locking down the computing environment to prevent unauthorised access or leaks. ERICA’s platform settings enforce a high-security, isolated ecosystem for data analysis. At all external access points, users must pass through rigorous authentication: a unique username/password plus a time-based one-time MFA code are required for login, and external access can be further restricted by IP address or device certificates if needed (for example, the UNSW ERICA’s web portal is only reachable from the university’s internal network or via VPN). Each user’s Workspace is a virtual Windows or Linux machine, which comes preconfigured with automatic antivirus and malware scanning that reports to a central log. Standard internet access from these workspaces is disabled for all users, meaning researchers cannot accidentally send data out of the Project Space by email or upload to cloud drives, etc.

System administrators have very limited inbound internet (through a tightly controlled proxy and only from allow-listed update servers for things like ERICA code updates, antivirus updates or software patching). Inbound internet access from within the research environment is similarly restricted using a “deny-all” proxy configuration, with curated exceptions for some sources such as R and Python package repositories, operating system updates, and required cybersecurity agents. This two-way isolation (no inbound access except through the portal, and no outbound access to the wider internet from within) creates a sealed analytic environment. Where AWS services require external connectivity (e.g. for patching or metadata access), curated exceptions or private endpoints are used to maintain functionality while adequately preserving security.

All administrator actions and maintenance operations are immutably logged so that even the system administrators cannot delete or modify log entries. The AWS cloud environment underpinning ERICA is configured with multiple network security layers, including VPC (Virtual Private Cloud) isolation, subnets that segregate sensitive components, and strict firewall rules. ERICA balances security and cost by using shared VPCs for core services with per-Project-Space peered VPCs for which transitive routing beyond their peer is not possible, subnets and refined route tables, along with selectively provisioned VPC endpoints and NAT (Network Address Translation) gateways to enable only necessary connectivity without incurring unnecessary costs.

Redundant safeguards ensure that if one control is accidentally misconfigured – unlikely due to the code-based configuration used - another will still block unintended access and data egress. Regular backups of ERICA data are performed to encrypted storage in multiple AWS availability zones (physically separated data centres in Sydney), protecting against data loss or corruption without exposing data outside the secure zone.

### Safe Outputs

Finally, ERICA enforces rigorous controls on safe outputs – the information that researchers remove from the TRE. Even after analysis is done, any result files (tables, statistical outputs, figures, code, etc.) must undergo approval before leaving the secure environment. When a researcher wishes to export analysis results, they submit the files via the ERICA Hub interface. The system requires the user to attest that they have applied appropriate Statistical Disclosure Control (SDC) techniques [12] and that the outputs do not contain identifiable information. The request then goes to the designated Project Controller for that Project Space. The Project Controller is a role within ERICA assigned by the project’s Chief Investigator or, in some cases, specified by a data custodian during project approval. The Project Controller reviews the files and ensures they comply with all data sharing conditions and privacy rules. Only after the Project Controller approves are the files released for download outside ERICA. This workflow ensures a second pair of eyes (with knowledge of the data) checks all outbound materials. ERICA also provides training and tools for SDC to help researchers produce aggregate results that are safe to release. Every file movement in or out, along with the approval records, is automatically logged and auditable. In fact, ERICA keeps a full copy archive of all files that are exported or imported via the Hub. This means that if there were any question later about what was shared, a complete audit trail is available and the actual files moved can be re-examined for safety. Audit logs (stored in AWS CloudWatch and CloudTrail) capture who requested and approved each egress, and cryptographic checksums of files ensure integrity (detecting if anything was altered). Because complete copies of all files entering and exiting each Project Space are retained, data custodians can also request to review outputs post-hoc, including random or directed spot-checks if they wish, adding an extra layer of oversight if desired. All users of each ERICA instance are made fully aware of these layers of oversight. Through these measures, ERICA maintains the confidentiality of research outputs: only non-sensitive results can leave, and all releases are tracked. This safe outputs control completes the end-to-end security of the TRE – from project inception to data import to analysis and finally to releasing results, every step is governed and monitored.

### Threat Model

Threat modelling works to identify, communicate, and understand threats and mitigations within the context of protecting something of value [13]. Space does not permit the fully articulated threat model which the ERICA architecture addresses to be presented here, but it can be summarised as follows:

- Legitimate, authorised users of ERICA instances (including end-users, particularly researchers, as well as IT system administrators, data custodians or data providers) are assumed to be good actors who are very unlikely to commit any act of malfeasance such as attempts to exfiltrate any sensitive data without approval, or otherwise attack or undermine privacy. This assumption is based on the very few reports of researchers anywhere behaving in this way, the career-limiting or career-ending consequences for researchers should such behaviour be detected, and the fact that the multiple layers of technical and administrative controls, checks and audit trails which the ERICA environment provides makes such detection likely.
- Even with thorough training, legitimate, authorised ERICA users are still human and may still be prone to making occasional inadvertent or innocent mistakes which might compromise privacy, should the ERICA environment permit such mistakes. Therefore, all aspects of the ERICA TRE should be designed to prevent such mistakes or make them very hard to make accidentally. In many cloud-based IT environments often found in universities and research institutions, it is very easy to inadvertently share access to files with a wider audience than intended. The ERICA environment specifically addresses this problem by completely removing all opportunities for sharing or providing access to sensitive data beyond the confines of each Project Space.
- In the unlikely event that an end-user account is compromised by an attacker (and hence a bad actor), the damage which that attacker is able to do must be limited by rigorous application of least privilege principles and requirements for data exports to be approved by a different account holder.
- All other actors, including all external actors, are assumed to be hostile and thus the architecture, technical cybersecurity controls and operating procedures must all be designed to minimise risks posed by such actors.

### Case Studies

To illustrate ERICA’s impact and capabilities, we present several case studies where the platform has been used to enable important research projects. These examples demonstrate how ERICA’s secure, code-driven environment supports real-world data analysis challenges in health and medical research, using a wide range of datasets including both unconsented, routinely collected data, and consented data collected through research studies and registries.

#### Case Study 1: CardiacAI (Cardiac Analytics and Innovation)

CardiacAI is a large-scale, multidisciplinary initiative aimed at improving cardiovascular outcomes by addressing one of the most persistent barriers in cardiac research—fragmented health data [14]. Led by a team of cardiologists and data scientists in New South Wales, the project integrates electronic medical records (EMRs) from multiple local health districts, including South Eastern Sydney and Illawarra Shoalhaven, with state-wide administrative datasets such as hospital admission and mortality records. By constructing a longitudinal view of patients’ cardiac care journeys, CardiacAI enables the application of advanced analytics and machine learning techniques to identify at-risk patients, optimise care pathways, and inform real-time clinical decision-making. The resulting integrated repository—unified, standardised, and research-ready—marks a significant step forward in overcoming institutional and data silos that have long hindered cardiovascular research.

ERICA has been central to enabling this transformation. Its secure, cloud-based architecture provided the trusted environment required to ingest, standardise, and link sensitive EMR data across institutions—giving data custodians the assurance needed to participate. Within ERICA, the CardiacAI team leveraged high-performance computing, including GPU-enabled virtual machines, to train deep learning models capable of predicting secondary cardiac events prior to hospital discharge. Importantly, ERICA’s flexibility allowed these powerful yet cost-intensive resources to be dynamically provisioned and decommissioned, aligning compute usage with analytical demand. This capability—difficult to achieve in traditional on-premise environments—has empowered CardiacAI to bridge clinical and technical domains, delivering actionable insights that are now being translated into live clinical dashboards. The project exemplifies how ERICA facilitates secure, high-impact research using sensitive health data to inform precision care at scale.

#### Case Study 2: AI Tools for Clinical Text

This project addressed a longstanding challenge in health data research: the ethical and legal barriers to using unstructured clinical text due to the presence of personally identifiable information. Led by a PhD researcher at UNSW in collaboration with the Australian Institute of Health and Welfare (AIHW), the project focused on developing advanced natural language processing (NLP) techniques to automatically de-identify sensitive content within electronic health records [15]. The team applied deep learning methods—including transformer-based models—to identify and remove personal identifiers from large volumes of free-text data including discharge summaries and clinical notes [16]. A custom-built web application within the secure environment enabled a human-in-the-loop workflow, allowing expert review and correction of the automated de-identification process to ensure high accuracy. The resulting system was capable of producing fully de-identified text suitable for secondary research use, offering a critical tool for unlocking insights from a previously underutilised data source.

ERICA played a foundational role in enabling this work by providing a secure, cloud-based environment that met the stringent privacy requirements associated with identifiable health data. The sensitive clinical texts remained entirely within the TRE, with no intermediate outputs permitted to leave until they were formally de-identified and approved. This assurance of containment and auditability was pivotal in securing access to the data and maintaining compliance with data governance policies. ERICA’s support for high-performance GPU computing also proved essential, facilitating the computationally demanding training of deep learning models on large-scale corpora. The project demonstrates ERICA’s unique value in supporting high-risk, high-reward applications of artificial intelligence in health research. It has since provided a blueprint for other custodians and researchers aiming to harness unstructured clinical data responsibly, highlighting ERICA as an enabling platform for privacy-preserving NLP in real-world settings.

#### Case Study 3: Rreal-World Evidence on Care Fragmentation Following Surgical Procedures

Understanding care fragmentation after major surgical procedures requires access to diverse, longitudinal health data that spans hospitals, emergency departments, and mortality records. This project employed ERICA to securely integrate and analyse large-scale, linked administrative datasets in order to evaluate post-operative outcomes following procedures such as aortic valve replacement [17] and joint arthroplasty [18]. By combining clinical registry data with routinely collected health service records, the project was able to capture patient trajectories beyond the index admission, uncovering patterns of unplanned readmissions, emergency presentations, and mortality that would have otherwise gone undetected.

ERICA played a central role in facilitating this work by providing a secure, cloud-based architecture that enabled the ingestion and analysis of fully identified patient-level data from multiple sources under strict governance protocols. The platform’s isolated Project Spaces allowed sensitive datasets—including state-level hospital and death records—to be linked and interrogated without risk of unauthorised access or data leakage. ERICA’s computational flexibility also supported the application of complex, longitudinal analysis workflows, including time-to-event modelling and cross-institutional data harmonisation. Through this infrastructure, the project not only identified gaps in registry-based outcome capture but also demonstrated how trusted research environments can enhance the utility of real-world data to inform surgical quality improvement and care coordination at scale.

## Discussion

ERICA represents a significant advancement in how we provision secure environments for data-intensive research. In the years since the platform was established, it has supported a wide range of projects (as illustrated by the case studies) that would have been difficult or impossible to conduct under traditional on-premise or less secure setups. By leveraging public cloud computing through a privacy-by-design lens, ERICA offers a combination of security, scalability, and flexibility that is novel in the TRE space. The cloud-based approach allows researchers to scale up compute resources on-demand (e.g. for machine learning tasks) and integrate new tools, all while the environment remains locked down according to strict governance policies. Each ERICA instance, governed by its host institution, maintains strong privacy and security controls compliant with local regulations – demonstrating that cloud TREs can meet the high standards required by data custodians and ethics committees. Indeed, using ERICA has, in some cases, helped expedite ethics approvals for projects: researchers can explicitly cite ERICA’s certified security measures and audit capabilities in ethics applications, reassuring committees that the data will be well-protected. This illustrates how an established TRE platform can reduce barriers to research by building trust with oversight bodies.

One of ERICA’s biggest strengths is the comprehensive governance and security framework baked into its design. This was validated by user feedback gathered through a recent survey and roundtable of ERICA users across different institutions. Respondents overwhelmingly highlighted security and data governance as the platform’s greatest strength, distinguishing it from other solutions. Features such as mandatory project approvals, role-based access, encryption, and audit logging instil confidence that sensitive data are handled appropriately. Users also appreciated ERICA’s flexibility – unlike some secure enclaves, ERICA allows a range of software, operating systems (Windows/Linux), and analytic approaches, and it can integrate with many AWS services for added functionality. This flexibility, combined with the inherent scalability of AWS, means ERICA can support everything from small projects to very large ones (such as those using billions of records or running large machine learning models) in a way that on-premise systems often cannot.

Some ERICA users with a Project Researcher role have both the skills and the requirements to make use of software development and operating system level services, upon which they build their own research data analysis systems and applications. Due to its architecture, the ERICA environment is able to safely accommodate such activities within the confines of each Project Space. The research analysis software so created may become part of their research outputs, abstracted from the underlying sensitive data. Most researcher end-users wish to use common statistical packages such as SAS, STATA, R, and increasingly, Python analytical software libraries. Many use a combination of tools. ERICA is able to provide researchers with this flexibility, within its security boundaries.

ERICA does not currently implement a centralised disclosure control model like those used by some TREs. Instead, responsibility for output review rests with the designated Project Controller—either appointed by the Chief Investigator or delegated by the data custodian. This model offers flexibility to accommodate institutional preferences and regulatory constraints. However, as with all TREs, there are potential scalability limitations for projects generating large volumes of outputs. To address this, ERICA allows institutions to adopt bespoke output checking models, such as waiving the requirement for output checking for projects using non-sensitive data, allocating specialist disclosure officers, or integrating automated Statistical Disclosure Control tools within the output workflow. In recognition of emerging risks, disclosure risk mitigation approaches for AI/ML, such as differential privacy and model inspection to detect inversion or membership inference vulnerabilities, are under active investigation within the ERICA program to support safe outputs from projects which are training or fine-tuning AI/ML models for use outside the ERICA environment.

At the same time, real-world use of ERICA has surfaced some limitations and challenges which point to areas for improvement. Cost has been noted as a concern – since ERICA runs on AWS, computing and storage expenses can accumulate, especially for long-running analyses or big data storage. Indicative costs vary based on workload patterns and configuration, but small-to-medium-sized projects typically incur cloud expenses of AUD 300–800 per month. Major cost drivers include large persistent storage volumes, per-user AWS WorkSpaces charges, and charges for bespoke compute and/or GPU servers which some research projects require, if these are not used judiciously and shut down when not required. Automated reminders prompting researchers to shut down such servers were implemented soon after the first ERICA instance was commissioned and had an immediately beneficial effect on containing costs of the more expensive server platforms required by some projects. Several users reported that AWS costs are a key issue. In response, the ERICA team is investigating cost-optimisation strategies, such as scheduling mechanisms to ensure all idle resources are shut down, using spot instances or other AWS cost-saving plans, and even the feasibility of a “cloud bursting” model where extremely large jobs could run on academic supercomputers or a government cloud, if security requirements can be met.

Another limitation raised has been the technical complexity of maintenance and upgrades. While the code-driven approach provides consistency, deploying updates to ERICA (e.g. new features or security patches) requires skilled AWS DevOps expertise. Smaller institutions noted difficulty in recruiting or affording staff with the necessary cloud and DevSecOps skills. This has led to discussions about offering a more centralised or managed service model in the future, or at least better documentation and tools to simplify upgrades. Additionally, certain technical features users desire – such as containerisation support (Docker), or support for additional development environments like Windows Subsystem for Linux – have not yet been fully incorporated into the code-as-infrastructure framework, although they can be provided as custom enhancements to particular ERICA Project Spaces. These are active areas of development, as the ERICA roadmap includes evaluating new AWS services (for example, AWS’s evolving container solutions, virtual desktop enhancements and preconfigured safe “landing zones” for enterprises) to extend functionality and/or reduce instance setup overhead while maintaining security.

Another challenge inherent to ERICA’s current design is its dependence on AWS. The decision to build on a single cloud vendor has clear benefits (tight integration with AWS security features, faster deployment using AWS-managed services), but it also means portability is limited. A technical investigation was conducted to see if ERICA could run on the Australian Nectar Research Cloud (an OpenStack-based academic cloud). The findings showed that migrating ERICA off AWS would require re-engineering many components from scratch due to lack of equivalent managed services and the need for 24/7 operations support. In essence, ERICA currently cannot be easily “lifted and shifted” to another cloud provider environment without significant redevelopment. If future government policy or cost pressures demand a non-AWS solution, ERICA’s design will need to adapt (perhaps by abstracting some services, or replicating the architecture on other cloud platforms). In the meantime, the benefits of AWS – including its continuous improvements and new service offerings – continue to enhance ERICA. The platform stays up-to-date with AWS’s releases (albeit new AWS features sometimes arrive later in the Sydney region than in the US).

Importantly, the ERICA approach has catalysed a culture change in how researchers approach sensitive data. Instead of moving data around to researchers’ local environments, with all of the attendant risks, researchers are effectively moving themselves into a secure environment where data already resides. This inversion – bringing the analysis to the data – is a cornerstone of modern data governance, and ERICA provides a workable, scalable way to do it. By demonstrating successful research outcomes in a TRE, ERICA has built confidence among stakeholders (data custodians, ethics boards, etc.) that valuable research and strong privacy protections can co-exist. It encourages more data custodians to make data available under these controlled conditions, knowing there is a proven mechanism to keep it safe. In turn, researchers gain access to richer datasets and can collaborate across institutions within TREs, accelerating discovery while upholding publictrust.

## Future Work

Future development of ERICA is centred on enhancing scalability, flexibility, and long-term sustainability. A key priority is the transition towards a modular, microservices-based architecture to simplify the deployment of new instances, reduce operational complexity, and optimise cloud resource utilisation. This approach is expected to support the introduction of a managed service model, allowing institutions with limited technical capacity to adopt ERICA without maintaining the full infrastructure stack.

On the technical front, ERICA is exploring the integration of on-demand high-performance computing (HPC) capabilities, leveraging AWS-native services or secure connectivity to national supercomputing resources. Fully integrated support for containerised workflows (e.g. Docker-based environments) are also under consideration to support modern data science pipelines while preserving strict isolation and audit controls.

In parallel, efforts are ongoing to evaluate multi-cloud and hybrid-cloud options, enabling future compatibility with platforms such as Microsoft Azure or Google Cloud for institutions with specific infrastructure needs or data residency requirements. This includes assessment of hybrid AWS deployment models, such as AWS Outposts and related management services (e.g. AWS Systems Manager), to support organisations with on-premise requirements while preserving ERICA’s security and governance model.

Work is also progressing towards meeting the accreditation standards required to host Commonwealth datasets such as MBS and PBS, which would significantly broaden ERICA’s research applications. Additional enhancements include exploration of privacy-preserving technologies—such as homomorphic encryption and secure multiparty computation—to enable secure, federated analysis between Project Spaces hosted in an ERICA TRE instance, or even between completely independent ERICA instances. Collectively, these initiatives position ERICA to remain at the forefront of trusted research environments, empowering researchers to work with sensitive data at scale while maintaining the highest standards of privacy, governance, and scientific integrity.

## Conclusion

ERICA provides a robust and secure foundation for data-intensive research, combining stringent privacy controls, mandatory training, and comprehensive audit logging to ensure high standards of data protection and integrity. Its code-driven orchestration and automated approval pathways streamline project setup and data access, while its multi-instance architecture enables collaboration and resource sharing across institutions. With ongoing enhancements—such as support for on-demand HPC—ERICA continues to evolve as a trusted, future-ready environment that advances multidisciplinary research while upholding public trust and ethical compliance.

## Data Availability

No data are associated with this manuscript.

## Abbreviations

AI:	Artificial Intelligence
AIHW:	Australian Institute of Health and Welfare
ARDC:	Australian Research Data Commons
AWS:	Amazon Web Services
CI:	Chief Investigator
CIFS:	Common Internet File System
DNS:	Domain Name System
EBS:	Elastic Block Store
EMR:	Electronic Medical Record
eHub:	ERICA Hub (data ingress/egress interface)
ERICA:	E-Research Institutional Cloud Architecture
GPU:	Graphics Processing Unit
HREC:	Human Research Ethics Committee
HPC:	High-Performance Computing
HTTPS:	HyperText Transfer Protocol Secure
IaC:	Infrastructure as Code
IP:	Internet Protocol
IRAP:	Infosec Registered Assessors Program
LDAPS:	Lightweight Directory Access Protocol over SSL
MFA:	Multi-Factor Authentication
MBS:	Medicare Benefits Schedule
NAT:	Network Address Translation
NLP:	Natural Language Processing
NSW:	New South Wales
OAIC:	Office of the Australian Information Commissioner
PBS:	Pharmaceutical Benefits Scheme
PSAF:	Privacy and Security Assessment Framework
SDC:	Statistical Disclosure Control
S3:	Simple Storage Service (AWS)
SMB:	Server Message Block
TLS:	Transport Layer Security
TRE:	Trusted Research Environment
UNSW:	University of New South Wales
USB:	Universal Serial Bus
URL:	Uniform Resource Locator
VPC:	Virtual Private Cloud
VPN:	Virtual Private Network
